# Effectiveness of eradication therapy for *Helicobacter pylori* infection in Africa: a systematic review and meta-analysis

**DOI:** 10.1186/s12876-023-02707-5

**Published:** 2023-03-07

**Authors:** Sintayehu Fekadu, Hizkel Engiso, Sisay Seyfe, Hisashi Iizasa, Ashebir Godebo, Serawit Deyno, Hironori Yoshiyama

**Affiliations:** 1 School of Laboratory Sciences, College of Medicine and Health Sciences, Hawassa University, P. O. Box 1560, Hawassa, Ethiopia; 2 School of Pharmacy, College of Medicine and Health Sciences, Hawassa University, P. O. Box 1560, Hawassa, Ethiopia; 3 Department of Biochemistry, College of Medicine and Health Sciences, Hawassa University, P. O. Box 1560, Hawassa, Ethiopia; 4 Department of Microbiology, Faculty of Medicine, Shimane University, Shimane, Japan; 5 Department of Soil Science, University of Saskatchewan, Saskatoon, SK Canada

**Keywords:** Africa, Eradication rate, First-line therapy, *H. pylori*

## Abstract

**Background:**

The effectiveness of *Helicobacter pylori* (*H. pylori*) eradication depends on the treatment protocol. This study investigates the *H. pylori* eradication rate in Africa using the best available evidence from databases.

**Methods:**

Databases were searched and results were pooled together. Heterogeneity between studies was assessed using I^2^ test statistics. Stata version 13 software was employed to compute the pooled eradication rate. In the subgroup analysis comparison, the finding is considered significant when the confidence intervals did not overlap.

**Results:**

Twenty-two studies from 9 African countries with a total population of 2,163 were included in this study. The pooled eradication rate of *H. pylori* was 79% (95% CI: 75%-82%), heterogeneity (I^2^ = 93.02%). In the subgroup analysis by study design, a higher eradication rate was reported from observational studies (85%, 95% CI: 79%-90%), compared to randomized control trials (77%, 95% CI: 73%-82%); by the duration of therapy, higher eradication rate was reported in 10-days regimen (88%, 95% CI: 84%-92%), compared to 7-days regimen (66%, 95% CI: 55%-77%); by country, the highest eradication rate was found in Ethiopia (90%; 95% CI: 87%-93%) and the lowest eradication rate was reported in Ivory Coast (22.3%; 95% CI:15%-29%); by type of *H. pylori* test, the highest eradication rate was reported when rapid urease test coupled with histology (88%, 95% CI: 77%-96%), and the lowest eradication rate was reported with histology alone (22.3%; 95% CI:15%-29%). Significant heterogeneity was observed with pooled prevalence (I^2^ = 93.02%, *P* < 0.000).

**Conclusions:**

In Africa, the first-line therapy showed a variable eradication rate for *H. pylori*. This study demonstrates the necessity to optimize current *H. pylori* treatment regimens in each country, taking into account the antibiotic susceptibility. Future RCT studies with standardized regimens are warranted.

**Supplementary Information:**

The online version contains supplementary material available at 10.1186/s12876-023-02707-5.

## Background

*Helicobacter pylori (H. pylori)* is a microaerophilic, Gram-negative, spiral-shaped motile bacterial pathogen that colonizes the gastric mucosa of approximately half of the world’s population. *H. pylori* infection is associated with gastritis, peptic ulcer, atrophic gastritis, gastric adenocarcinoma, and mucosa-associated lymphoid tissue lymphoma (MALT). The presentation of a range of clinical conditions is primarily determined by bacterial virulence, host genetics, and the individual’s lifestyle [1–4]. The prevalence of *H. pylori* infection varies globally, with Africa being the highest infection rate [5]. The bacterium is primarily acquired during early childhood under low socioeconomic conditions and close family contact [6].

According to the Maastricht VI/Florence consensus report 2022, individuals with or without clinical evidence of *H. pylori* infection are recommended to receive first-line eradication therapy to prevent the development of infection-associated complications, such as gastritis and cancer [7]. Moreover, large-scale eradication of *H. pylori* in a population reduced the incidence and mortality of gastric cancer [8]. In light of this, guidelines have been developed as a national or regional first-line eradication protocol that consists of different antibiotic combinations, including triple therapies, bismuth-free therapies (sequential, concomitant, or hybrid regimens), and bismuth-based quadruple therapy [9]. The effectiveness of eradication therapy has been assessed based on the pre-protocol analysis and categorized as excellent (≥ 95% success), good (≥ 90% success), borderline acceptable (85–89% success), or unacceptable (< 85% success) [10]. The presence of *H. pylori* resistance to one or more antimicrobial agents or poor medication adherence increases the likelihood of treatment failure, even with excellent regimens.

*H. pylori* eradication rate differs in different settings based on the type of regimen employed, duration of therapy, and local antibacterial susceptibility pattern. According to a recent systematic review and meta-analysis, first-line treatment had a 98% global *H. pylori* eradication rate, with a subcontinental success rate of 98% in Asia, 94% in Africa, 94% in Europe, 93% in South America, and 84% in North America. In this report, five African countries with a total of 7 studies comprising 1021 patients were included, Morocco (*n* = 3), Egypt (*n* = 1), Kenya (*n* = 1), Nigeria (*n* = 1), and Tunisia (*n* = 1) [11].

However, there is no pooled eradication rate consisting of observational and randomized controlled trials for *H. pylori* infection in Africa. However, small-scale studies were reported in different countries in Africa. Therefore, African studies differ in study settings, methodology, and other characteristics. In addition, no systematic review or meta-analysis has been conducted on the eradication rate of *H. pylori* infection in Africa. Therefore, we have undertaken a systematic review to determine the eradication rate of *H. pylori* in Africa using previously published articles.

## Methods

### Databases and search strategy

**Fig. 1:**
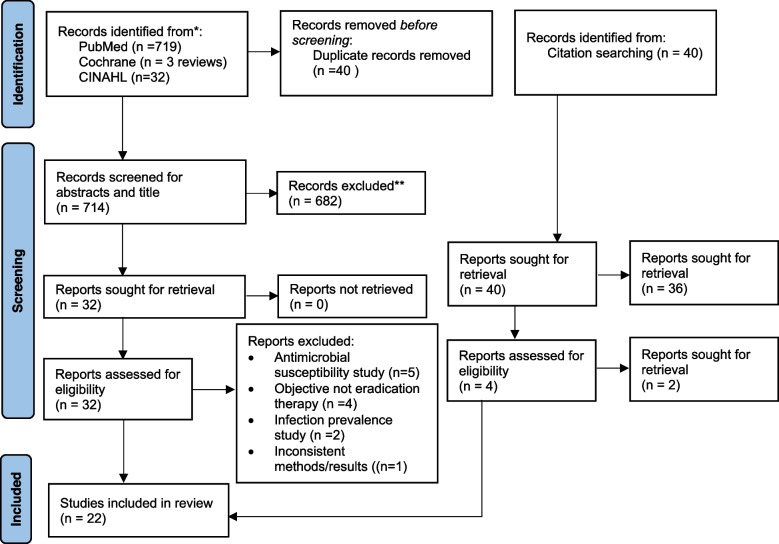
PRISMA flow chart of studies selection

PubMed, Google Scholar, Hinari, Scopus, and the Directory of Open Access Journals (DOAJ) were searched to identify potential articles on *H. pylori* eradication in Africa. The search was conducted following PRISMA guidelines and checklists [12], Fig. 1.

### Quality assessment

The quality of included studies was assessed by using a revised Cochrane risk-of-bias tool for randomized trials (RoB2) and Risk Of Bias in Non-Randomized Studies-of Interventions (ROBINS-I) (Supplementary files 1 and 2). The authors independently assessed the quality of each study, and a consensus was reached on twenty-two studies conducted in nine African countries.

### Data extraction

Data were extracted into a customized Microsoft excel spreadsheet. The characteristics of extracted data in each study include: first author name, year of publication, country of study, study design, number of study participants, characteristics of study participants (naïve or nan-naïve), laboratory methods for *H. pylori* positivity test, number of *H. pylori-*positive participants, *H. pylori* eradication regimen, duration of follow up, laboratory methods for *H. pylori* eradication confirmation, and number of *H. pylori* eradicated individuals. In addition, graphs of the summary of the risk of bias were developed using RevMan 5.3 (Cochrane Informatics and Knowledge Management Department, London, UK).

### Data analysis

Statistical analyses were conducted using Stata version 13.0 (StataCorp, LP, college station, TX). The eradication rate values were pooled using the *metaprop* command in Stata. The heterogeneity of the studies was assessed using the I^2^ statistic, and significance was declared at I^2^ > 50% and Q-test (*p* < 0.10). Because of high heterogeneity among the studies, the random-effects model (REM) was used to estimate the pooled proportion and 95% CIs using the DerSimonian and Laird methods. The Freeman-Turkey double arcsine transformation was used to avoid missing proportions near or at 0 and 1 from the meta-analysis. Subgroup analysis was done by study design, country, laboratory tests for *H. pylori* infection, eradication regimen, type of regimen analysis, characteristics of the study population, follow-up duration, and tests employed to confirm eradication. The presence of publication bias was tested using Egger’s test. Forest plots and tables were constructed to display the individual studies and pooled results.

### Publication bias and sensitivity analysis

A funnel plot was drawn to evaluate the potential for publication bias. The funnel plots’ gap suggests potential publication bias. In addition, Egger’s regression asymmetry tests were used to assess publication bias, with *p* < 0.05 considered to indicate potential publication bias. Finally, sensitivity and leave-one-out analysis were done to evaluate the prime determinant of the pooled eradication rate and to detect the possible causes of heterogeneity between studies.

## Results

### Characteristics of included studies

Twenty-two studies from nine African countries with a total population of 2,163 met the inclusion criteria of the meta-analysis. These studies were published articles from 1992 to 2020, and the number of articles by country is indicated in Table 1. The detailed characteristics of included studies are presented in Table 2. Among the included studies, 8 were observational, and 14 were randomized control trials (RCT). Except for Abd-Elsalam et al., 2016, all study participants were newly diagnosed cases with gastrointestinal disorder. Twelve studies used multiple tests to detect *H. pylori,* while 10 employed a single test to declare *H. pylori* infection. Eighteen studies employed a single test, and 4 studies used multiple tests to prove *H. pylori* eradication. The *H. pylori* eradication rates in the qualified studies ranged from 22.3% to 90%.

**Table 1:** Number of articles included in the study by country

Country	Number of articles	Reference
Egypt	7	[13–19]
Morocco	4	[20–23]
South Africa	4	[24–27]
Algeria	2	[28, 29]
Ethiopia	1	[30]
Nigeria	1	[31]
Tanzania	1	[32]
Kenya	1	[33]
Ivory Coast	1	[34]

**Table 2:** Lists and characteristics of included 22 studies

Authors	Study Type	Country	*H. pylori* Positive	Eradicated	Eradication rate, %	Test method	Regimen	Duration (days)	Outcome measure (weeks)	Confirmed test
Elkhodary et al., 2020 [14]	RCT	Egypt	33	21	63.6	FAT	DLA	7	4	FAT
Farhoud_1 et al., 2020 [15]	RCT	Egypt	30	19	63.3	RUT	LAC	14	6	UBT
Zeriouh_1 et al., 2020 [23]	RCT	Morocco	124	84	67.7	UBT or H	PPI + A	14	6	UBT
Gebeyehu et al., 2019 [30]	PS	Ethiopia	421	379	90.0	FAT	OAC	14	4	FAT
Hassan et al., 2019 [17]	RCT	Egypt	50	31	62.0	FAT	OAC	14	4	FAT
Jaka et al., 2019 [32]	PS	Tanzania	210	145	69.0	FAT	PPICM/A	10	5	FAT
Moubri_1 et al., 2019 [28]	RCT	Algeria	55	39	70.9	C + H + UBT	OAC	7	8	UBT
Moubri et al., 2019 [29]	PS	Algeria	101	79	78.2	C or H + RUT	PPIAC	14	8	UBT
Shehata_1 et al., 2017 [18]	RCT	Egypt	112	106	94.6	FAT + H	OCN	14	6	FAT
Abd-Elsalam et al., 2016^a^ [13]	RCT	Egypt	94	83	88.3	FAT	OLDN	14	6	FAT
Hanafy et al., 2016 [16]	PS	Egypt	248	169	68.1	FAT	LAC	14	4	FAT
Abou Saif et al., 2015 [19]	RCT	Egypt	18	17	94.4	FAT	5OA,5OLM	10	4	FAT
Doffou et al., 2015 [34]	RCT	Ivory Coast	64	18	28.1	H	OAM	7	4	H
Onyekwere et al., 2014 [31]	RCT	Nigeria	29	25	86.2	UBT	RAC	10	4	UBT
Benajah_1 et al., 2013 [20]	PS	Morocco	204	156	76.5	RUT + H + C	OAM/C	7	12	UBT
Laving_1 et al., 2013 [33]	RCT	Kenya	45	22	48.9	H	OAC	10	6	FAT
Seddik_1 et al., 2013 [22]	RCT	Morocco	129	116	89.9	H	5OA,OCT	10	10	UBT
Lahbabi et al., 2013 [21]	RCT	Morocco	103	73	70.9	H or PCR	PPIAM	14	12	FAT
Wong et al., 2000 [27]	PS	South Africa	22	19	86.4	H + RUT + UBT	OAC	7	4	H + UBT
Louw_1 et al., 1998_a [25]	PS	South Africa	24	22	91.7	RUT + H	LAC	7	4	RUT + H
Louw_1 et al., 1998_b [26]	PS	South Africa	30	26	86.7	RUT + H + C	PAC	7	4	RUT + H + C
Louw_1 et al., 1992 [24]	RCT	South Africa	17	10	58.8	RUT + C + H	S + B + OA	14	4	RUT + H + C

*FAT* Fecal antigen test, *RCT* Randomized controlled trial, *PS* Prospective study, *PP* Per protocol, *ITT* Intention to treat, *UBT* Urea breath test, *H* Histology, *RUT* Rapid urease test, *C* Culture, *PCR* Polymerase chain reaction, *OAC* Omeprazole + Amoxicillin + Clarithromycin, *OCM* Omeprazole + Clarithromycin + Metronidazole, *OAM* Omeprazole + Amoxicillin + Metronidazole, *LAC* Lansoprazole + Amoxicillin + Clarithromycin, *PAC* Pantoprazole + Amoxicillin + Clarithromycin, *RAC* Rabeprazole + Amoxicillin + Clarithromycin, *OCN* Omeprazole + Clarithromycin + Nitazoxanide, *OACS* Omeprazole + Amoxicillin + Clarithromycin + Simvastatin, *OLDN* Omeprazole + Levofloxacin + Doxycycline + Nitazoxanide, *DLA* Dexolansoprazol + Levofloxacin + Amoxicillin *OCT* Omeprazole + Clarithromycin + Tinidazole

^a^Study participants were non-naïve

**Fig. 2:**
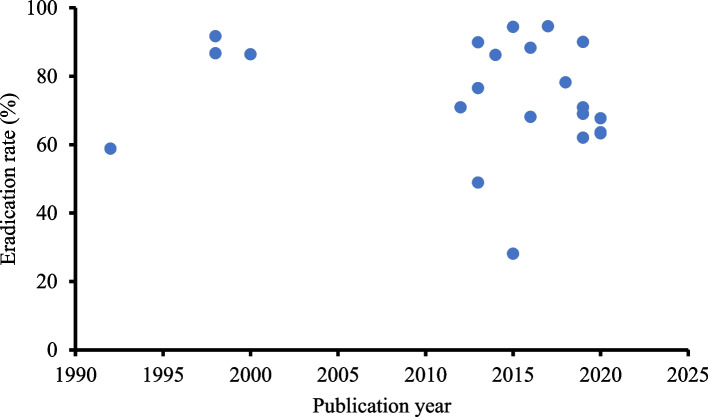
Trends in *H. pylori* eradication rate in Africa

The eradication rate and the retrieved studies varies with time. Trend analysis is done to explore the time effect of the study using a scatter plot as indicated in Fig. 2. The trend analysis shows that from 2000 to 2010 there is no eradication study and no variability observed with time.

### Pooled eradication rate of *H. pylori*

**Fig. 3:**
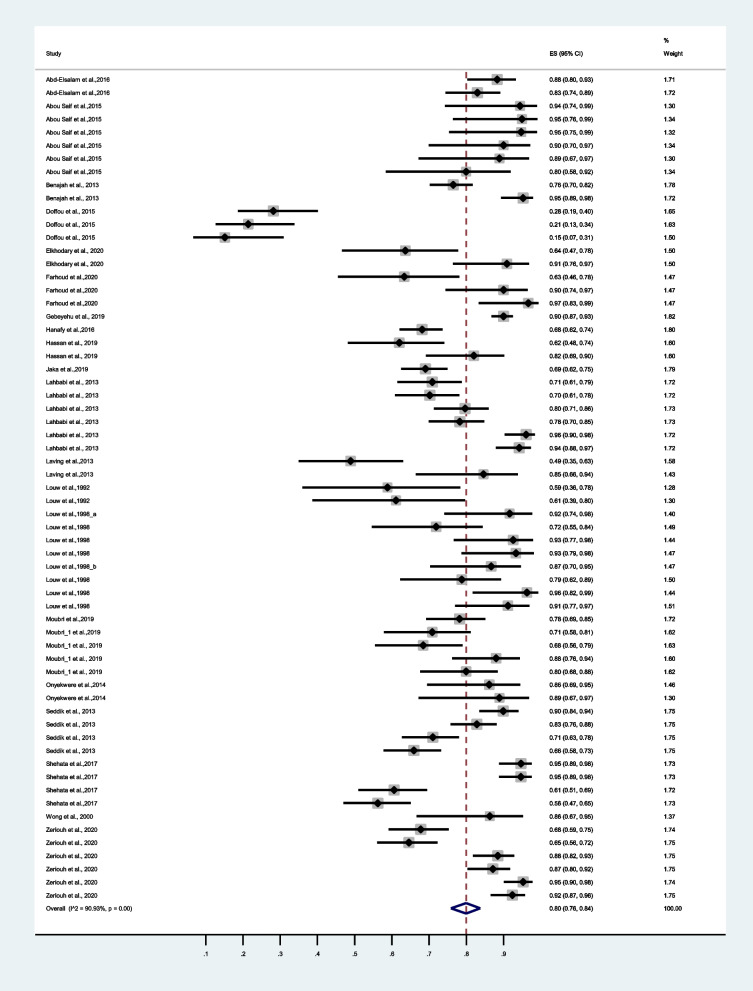
Forest plots of the pooled eradication rates of *Helicobacter pylori* infection by first-line standard therapy in Africa from 22 studies

**Fig. 4:**
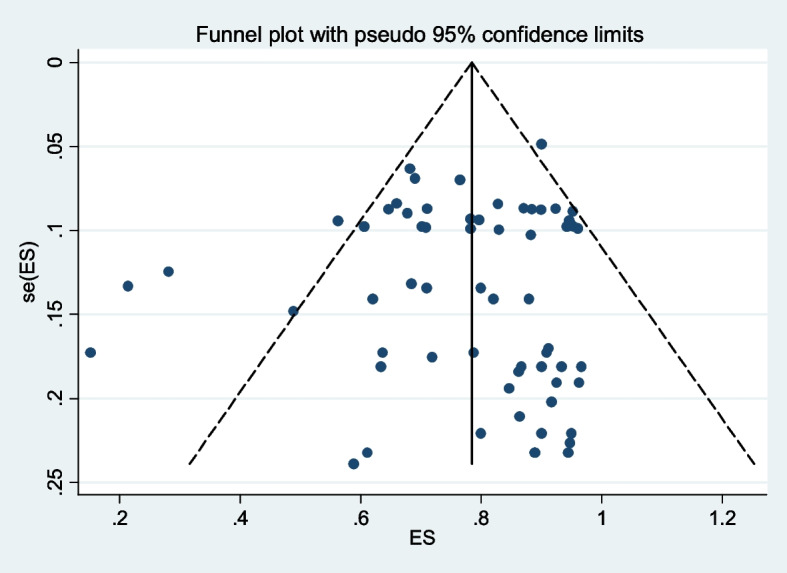
Funnel plot showing absence of publication bias with no small study effects, *p* = 0.672. Publication bias assessment funnel plot; Egger’s regression test (*p* = 0.672)

A total of 2,163 people tested positive for *H. pylori* in Africa. Of which 1,659 confirmed eradication following first-line eradication therapy in the period under review. Our meta-analysis revealed pooled eradication rate of 79% (95% CI: 75%-82%), I^2^ = 93.02% (Fig. 3). Moreover, the funnel plot for publication bias supported Egger’s regression (*p* = 0.672) test, which showed no significant publication bias (Fig. 4).

### Subgroup eradication rate of *H. pylori*

Subgroup analyses were conducted by country, study design, type of analysis, study population, duration of therapy, outcome measures and regimen. The pooled data were from nine countries. In addition, more studies were conducted in Egypt, which showed an eradication rate of 82%, and almost all countries showed a similar eradication rate except Ivory Coast (22.3%) (Table 3).

**Table 3:** The subgroup analysis of included studies by country, study design, type of analysis, study population, duration of therapy, outcome measures and regimen from 22 studies in Africa

Subgroup	Eradication rate (%)	95% CI (%)	I^2^ (%)
Country			
Egypt (*n* = 7)	82	(77–88)	89.85
Morocco (*n* = 4)	82	(77–87)	92.2
South Africa (*n* = 4)	86	(80–92)	60.17
Algeria (*n* = 2)	78	(71–85)	53.33
Ethiopia (*n* = 1)	90	(87–93	-
Nigeria (*n* = 1)	87	(78–97)	-
Tanzania (*n* = 1)	69	(62–75)	-
Kenya (*n* = 1)	68	(58–78)	-
Ivory Coast (*n* = 1)	22.3	(15–29)	-
Study design			
PS	85	(79–90)	88.76
RCT	77	(73–82)	93.84
Types of analysis			
PP	78	(73–82)	94.18
ITT	81	(76–86)	88.67
Study population			
Naïve	79	(75–82)	93.24
Non-naïve	86	(81–91)	-
Duration of therapy (days)			
14	80	(74–85)	88.73
10	88	(84–92)	83.49
7	66	(55–77)	91.52
Outcome measures (weeks)			
4	78	(71–84)	91.2
6	81	(73–88)	92.7
8	81	(72–88)	69.4
12	84	(76–91)	90.7
H. pylori confirmed test			
FAT	82	(76–87)	90.23
UBT	83	(78–88)	88.5
H	22.3	(16–30)	-
UBT + H	86	(67–95)	-
RUT + H	88	(77–96)	54.44
RUT + H + C	82	(69–92)	69.54
FAT + RUT + H + C	77	(68–85)	59.28
Regimen			
OLDN	86	(80–90)	
5OA, 5OLM	95	(84–100)	
5OA, 5OCM	95	(91–99)	
14OCM	84	(71–95)	
OAM/C	76	(70–82)	
OAM	67	(27–96)	
OAC	66	(50–80)	
OCM	44	(23–67)	
DLA	79	(68–88)	
LAC	81	(69–91)	
5LA, LCT	90	(74–97)	
7LA, LCT	97	(83–99)	
OACS	82	(69–90)	
PPIAM	71	(64–77)	
PPIAC	79	(74–83)	
5PPIA, PPIMC	95	(92–98)	
5OA, OCT	85	(66–94)	
S + B + OA	59	(36–78)	
S + OA	61	(39–80)	
PAC	89	(81–95)	
RAC	87	(76–96)	
5OA, OCT	86	(82–90)	
OCN	95	(91–97)	
PPI + A	66	(60–72)	
5PPI + A, PPI + ACM	88	(84–92)	
OACM	94	(91–97)	

*PS* Prospective study, *RCT* Randomized control trial, *PP* Per protocol, *ITT* Intention to treat, *FAT* Fecal antigen test, *UBT* Urea breath test, *H* Histology, RUT Rapid urease test, *C* Culture, *OAC* Omeprazole + Amoxicillin + Clarithromycin, *OCM* Omeprazole + Clarithromycin + Metronidazole, *OAM* Omeprazole + Amoxicillin + Metronidazole, *LAC* Lansoprazole + Amoxicillin + Clarithromycin, *PAC* Pantoprazole + Amoxicillin + Clarithromycin, *RAC* Rabeprazole + Amoxicillin + Clarithromycin, *OCN* Omeprazole + Clarithromycin + Nitazoxanide, *OACS* Omeprazole + Amoxicillin + Clarithromycin + Simvastatin, *OLDN* Omeprazole + Levofloxacin + Doxycycline + Nitazoxanide, *DLA* Dexlansoprazole + Levofloxacin + Amoxicillin *OCT* Omeprazole + Clarithromycin + Tinidazole

## Discussions

The 22 studies included in our analysis showed the pooled eradication rate in Africa was estimated to be 79% (95% CI: 75–82). This overall eradication rate is lower than reports from Ethiopia (90%), Nigeria (87%), South Africa (86%), Egypt (82%), Morocco (82%), and higher than reports from Tanzania (69%), Kenya (68%) and Ivory Coast (22.3%). These differences might be attributed to methods employed to diagnose *H. pylori*, type of eradication regimen and duration of therapy, local *H. pylori* pretreatment resistance,and drug adherence, as stated by previous reports [35–39].

Trend analysis in *H. pylori* eradication from the 22 studies showed that presence of few studies in the year between 1990s and 2000. There is no relevant study between the year 2000 and 2010. The trend also showed that more publications coming out since 2011. This trend analysis showed that decrease in *H. pylori* eradication rate. Similar studies indicated that decreasing trends of eradication rate for *H. pylori* over the years [40]. On the other hand, *H. pylori* eradication rate consistent in study conducted for a decade [41]. The decrement in eradication rate could be attributed to increasing resistance due to increased antibiotic exposure.

The eradication rate for *H. pylori* varies in different regions of the world. The overall success of eradication depends on the choice of eradication regimen, duration of the treatment, and local and regional antibiotic resistance pattern of *H. pylori*. The World Gastroenterology Organization (WGO), in its 2023 guideline, recommended the minimum acceptable eradication rate greater than 80% on an intention-to-treat basis using PPI-clarithromycin plus amoxicillin in areas where clarithromycin resistance is low or moderate [42]. Determining the national and regional eradication rate is fundamental to establish an appropriate eradication protocol for *H. pylori* infection. Estimating the effectiveness of *H. pylori* eradication is difficult since factors like pretreatment antibiotic resistance have a profound effect [43–46]. Some studies consider distinct *H. pylori* diagnosis or eradication confirmation tests and employ different eradication regimens and/or duration based on the local guideline on the *H. pylori* treatment [47–49]. In the regional context, *H. pylori* treatment in Africa largely depends on an empirical approach despite the highest infection rate in the world [50, 51].

In the subgroup analysis by country, the highest eradication rate of 90% was from Ethiopia, and the lowest was 22.3% from Ivory Coast, as shown in Table 3. The eradication rate depends on the sensitivity and specificity of tests that detect *H. pylori*. The sensitivity and specificity of diagnostic or screening techniques depend on the laboratory techniques employed, personal skill to perform the test, and even the brand of reagents and facility standard. In the study from Ivory Coast, the *H. pylori* pre-and post-eradication detection was based on histological examination of gastric biopsy, which is less sensitive than conventional techniques such as urease test, anti-*H. pylori* antibody test, and PCR detection of bacterial genome. This finding is consistent with studies showing that treatment efficacy varies with *H. pylori* detection techniques [52–55].

This analysis presented the cumulative eradication rate for Africa and identified factors associated with eradication. In addition, the study included a sub-group analysis of differences in study design, country, treatment regimen, type of analysis, duration of eradication therapy, weeks of outcome measure and tests employed for *H. pylori* diagnosis and eradication confirmation. Africa contains 54 countries; however, this study has picked up reports only from 9 countries. Moreover, one-third of data are reported from Egypt. The fact might influence the generalization of our findings. Thus, eradication studies in Africa are so rare that more research must be promoted. There are so many regimen subgroups and result could be difficult to comprehend. But the general finding is that there is not significant variability among the regimen subgroups.

The current study included from observational to randomized control trails whereas the previous systematic review and network meta-analysis included only randomized controlled trials [11]. In Africa there is only very few RCT and only 7 articles are included. As a result, paucity of literature landscape in Africa, the current study has included observational study in addition to RCT. This has given a better understanding of eradication rate in Africa than the previous global meta-analysis. The study suffers from heterogeneity and there is no generalizable finding.

## Conclusion

In Africa, the first-line therapy showed variable eradication rate for *H. pylori*. This study demonstrates the need to reassess antibiotic susceptibility in each country and optimize current *H. pylori* treatment regimens. Antibiotic susceptibility of *H. pylori* should be investigated in each nation of Africa. Although gastrointestinal disorders and associated *H. pylori* infections are common problems in Africa, less attention is given to translate the efficiency of eradication and improve the eradication regimen. Future RCT studies with standardized regimens are required.

## Supplementary Information

**Additional file 1:** **Supplementary file 1.** ‘Checklist risk-of-bias assessment for randomized trials (RoB2)’.

**Additional file 2:** **Supplementary file 2.** 'Checklist risk-of-bias in non-randomized studies of intervention (ROBINS-I).'

**Additional file 3:** **Supplementary file 3.** Databases and search strategy.

## Data Availability

All data generated or analyzed are included in the result of the manuscript and its supplementary files.

## Abbreviations

CI: Confidence interval
*H. pylori*: *Helicobacter pylori*
OAC: Omeprazole + Amoxicillin + Clarithromycin
OCM: Omeprazole + Clarithromycin + Metronidazole
OAM: Omeprazole + Amoxicillin + Metronidazole
LAC: Lansoprazole + Amoxicillin + Clarithromycin
PAC: Pantoprazole + Amoxicillin + Clarithromycin
RAC: Rabeprazole + Amoxicillin + Clarithromycin
OCN: Omeprazole + Clarithromycin + Nitazoxacine
OACS: Omeprazole + Amoxicillin + Clarithromycin + Simvastatin
OLDN: Omeprazole + Levofloxacin + Doxycycline + Nitazoxanide
DLA: Dexlansoprazole + Levofloxacin + Amoxicillin
OCT: Omeprazole + Clarithromycin + Tinidazole
PS: Prospective study;
RCT: Randomized control trial
PP: Per protocol
ITT: Intention to treat
FAT: Fecal antigen test
UBT: Urea breath test
H: Histology
RUT: Rapid urease test
C: Culture
