# Supplementary material for: Effectiveness of eradication therapy for Helicobacter pylori infection in Africa: a systematic review and meta-analysis
Source: BMC Gastroenterol. 2023 Mar 7;23:55. doi: 10.1186/s12876-023-02707-5 (PMC9990047; doi:10.1186/s12876-023-02707-5)
Supplement: Supplementary file 3 — Additional file 3: Supplementary file 3. Databases and search strategy. [file 12876_2023_2707_MOESM3_ESM.docx]

Supplementary file 3

**Databases and search strategy**

PubMed, Google Scholar, Hinari, Scopus, and the Directory of Open Access Journals (DOAJ) were searched to identify potential articles on *H. pylori* eradication in Africa. The search was conducted following the PRISMA guideline and checklists ([12], Fig. 1). To search PubMed, the following terms were combined using MeSH (Medical Subject Headings) and Boolean operators; “*Helicobacter pylori*” “OR” “*H. pylori*” AND “eradication therapy” OR “treatment failure” OR “triple therapy” OR “quadruple therapy” OR “antimicrobial resistance” OR “antibacterial resistance” OR “antibiotic resistance” OR “efficacy” OR “effectiveness” OR “treatment” AND “Africa” OR “Algeria” OR “Angola” OR “Benin” OR “Botswana” OR “Burkina Faso” OR “Burundi” OR “Cameroon” OR “Canary Islands” OR “Cape Verde” OR “Central African Republic” OR “Chad” OR “Comoros” OR “Congo” OR “Democratic Republic of Congo” OR “Djibouti” OR “Egypt” OR “Equatorial Guinea” OR “Eritrea” OR “Ethiopia” OR “Gabon” OR “Gambia” OR “Ghana” OR “Guinea” OR “Guinea Bissau” OR “Ivory Coast” OR “Cote d’Ivoire” OR “Jamahiriya” OR “Kenya” OR “Lesotho” OR “Liberia” OR “Libya” OR “Madagascar” OR “Malawi” OR “Mali” OR “Mauritania” OR “Mauritius” OR “Mayotte” OR “Morocco” OR “Mozambique” OR “Namibia” OR “Niger” OR “Nigeria” OR “Rwanda” OR “Sao Tome” OR “Senegal” OR “Seychelles” OR “Sierra Leone” OR “Somalia” OR “South Africa” OR “South Sudan” OR “St Helena” OR “Sudan” OR “Swaziland” OR “Tanzania” OR “Togo” OR “Tunisia” OR “Uganda” OR “Western Sahara” OR “Zaire” OR “Zambia” OR “Zimbabwe” OR “Central Africa” OR “Central African” OR “West Africa” OR “West African” OR “Western Africa” OR “Western African” OR “East Africa” OR “East African” OR “Eastern Africa” OR “Eastern African” OR “North Africa” OR “North African” OR “Northern Africa” OR “Northern African” OR “South African” OR “Southern Africa” OR “Southern African” OR “sub Saharan Africa” OR “sub Saharan African” OR “subSaharan Africa” OR “subSaharan African”. The search included articles published in both English and non-English language. Google Translate was used to determine a publication’s eligibility for data extraction if it was written in a language other than English. To minimize bias and reduce the chance of missing studies, each author participated in extracting relevant articles from the different databases. The search results were combined into EndNote 20 (Clarivate Analytics USA). Articles were selected based on predefined inclusion criteria. This study included articles with the following characteristics: Observational or Randomized Clinical Trials (RCT), original journal articles, articles that reported *H. pylori* eradication therapy on human subjects, and articles that reported *H. pylori* eradication therapy in Africa. Duplicate studies, studies that reported *in vitro* anti-*H. pylori* susceptibility testing, studies that primary objective was not eradication therapy, and studies with inconsistent methodology or results were excluded. All articles that met the eligibility requirements and were published up through March 2022 were included. Two-stage selection of the articles was conducted. During stage one, the titles, abstracts, tables and figures of all retrieved articles were reviewed, and those articles that addressed the study question were deemed eligible for further review. However, those articles that did not satisfy our selection criteria were dropped. During stage two, the eligible articles were reviewed in full detail, and data mining was performed.
